# Subwavelength Nanostructuring of Gold Films by Apertureless Scanning Probe Lithography Assisted by a Femtosecond Fiber Laser Oscillator

**DOI:** 10.3390/nano8070536

**Published:** 2018-07-16

**Authors:** Ignacio Falcón Casas, Wolfgang Kautek

**Affiliations:** Department of Physical Chemistry, University of Vienna, Währinger Strasse 42, Vienna A-1090, Austria

**Keywords:** near-field, femtosecond laser, nanolithography, subwavelength, tip-enhancement, AFM

## Abstract

Optical methods in nanolithography have been traditionally limited by Abbe’s diffraction limit. One method able to overcome this barrier is apertureless scanning probe lithography assisted by laser. This technique has demonstrated surface nanostructuring below the diffraction limit. In this study, we demonstrate how a femtosecond Yb-doped fiber laser oscillator running at high repetition rate of 46 MHz and a pulse duration of 150 fs can serve as the laser source for near-field nanolithography. Subwavelength features were generated on the surface of gold films down to a linewidth of 10 nm. The near-field enhancement in this apertureless scanning probe lithography setup could be determined experimentally for the first time. Simulations were in good agreement with the experiments. This result supports near-field tip-enhancement as the major physical mechanisms responsible for the nanostructuring.

## 1. Introduction

Optical methods in lithography are limited by Abbe’s diffraction limit. Apertureless scanning probe nanolithography assisted by a laser is a method able to overcome this limitation [1,2,3,4,5,6,7,8,9]. In this technique, a sharp scanning probe microscope (SPM) tip is placed a few nanometres above the surface of a substrate. The tip is irradiated by a laser and a strong enhanced field may be generated in the proximity of the apex of the tip. The evanescent near-field decays exponentially in both lateral and vertical axes, which leads to a confinement of the electromagnetic field. This may surpass the modification fluence threshold of the substrate. The combination of tip-enhancement and confinement can be employed to produce sub-wavelength surface nanostructuring. However, although near-field enhancement has been predicted in simulations [10,11,12,13] and observed experimentally [14,15,16,17,18,19,20], a number of unresolved issues still exist. An example is how much thermal effects are contributing. On one hand, a laser-irradiated tip can reach a high temperature [21,22,23], leading to the destruction or modification of the tip itself. Even if the tip is not modified, heat transfer from the hot tip to the substratee—either by conduction or radiation—may lead to the melting of the substrate. Recent research has found huge near-field heat transfer coefficient values, whose origin has not been clarified yet [24,25,26]. On the other hand, laser irradiation can produce a fast bending and expansion of cantilevers due to thermal diffusion, leading to mechanical indentation or scratching of the surface of the substrate [23]. This problem can be handled by using low spring constant cantilevers [4] or keeping the tip in non-contact mode [8].

Femtosecond lasers have demonstrated excellent performance in material ablation because the heat affected zone can be minimized to a few nanometres and can avoid laser–plasma interactions completely. Continuous-wave lasers have also been employed in scanning probe optical lithography, but the use of ultrashort pulsed lasers allows for reducing the average power applied to the tip, while producing very high intensities. Although these high intensities can be beneficial regarding substrate structuring, laser intensities above the MW/cm${}^{2}$ level might lead to tip damage. Special care has to be taken with metal-coated tips, as it has been reported in apertureless scanning near-field microscopy (aSNOM) experiments [27].

Apertureless scanning probe near-field lithography assisted by femtosecond laser has demonstrated subwavelength surface structuring of metal and polymer films, reaching linewidths down to 10–15 nm [4,8]. These experiments were performed with Ti:Sa laser sources, at laser wavelengths λ≈ 790 nm. Theoretical calculations have shown that, under some conditions, longer laser wavelengths might lead to higher near-field enhancement [28,29]. A combination of high repetition rate and low energy per pulse helps to reduce thermomechanical instabilities of the tip-cantilever [30].

In the present study, apertureless scanning probe near-field lithography was employed to write nanofeatures on the surface of gold films. A home-built femtosecond Ytterbium-doped fiber laser oscillator based on a novel design [31] was applied. A repetition rate of 46 MHz and a low pulse energy was chosen to allow a better thermomechanical equilibrium of the cantilever in contrast to laser sources with kHz repetition rates and higher energy per pulse [30]. The generated subwavelength grooves exhibited typical linewidths of 40–60 nm, down to 10 nm, thus surpassing the diffraction limit. The near-field enhancement could be determined experimentally for the first time, and was compared with simulations.

## 2. Materials and Methods

We employed a scanning probe microscope NTEGRA (NT-MDT, Moscow, Russia) working in atomic force microscope (AFM) contact mode to scan the surface of the samples. AFM measurements were done in ambient conditions. A closed-loop configuration of the scanner stage was enabled to increase the lateral positioning accuracy. We used silicon probes with a radius of curvature of 10 nm (CSG10, NT-MDT) at the apex of the tip. Contact mode low spring constant cantilevers were chosen to avoid mechanical deformation of the gold films. The measured resonance frequency of the cantilever was 27 KHz, 30 μm width and 225 μm length. By using these values, and following the Sader’s method [32], we obtained a spring constant of the cantilever k = 0.4 N/m. We determined the load force applied against the sample from the spring constant value of the cantilever. We performed force spectroscopy on a silicon calibration grating (TGZ1, NT-MDT, Moscow, Russia) in order to obtain a linear relationship between cantilever deflection (in nA) and cantilever height (in nm). A deflection setpoint value of 2 nA was chosen when scanning in contact mode, corresponding to a load force of F${}_{load}$ ≈ 20 nN. This low value of the load force was chosen to avoid the possibility of mechanical surface modification. The scanning probe microscope data were evaluated with the software Gwyddion.

Gold films were prepared by thermal evaporation. Two films were deposited on mica (15 and 30 nm thickness) and another on a glass substrate (30 nm thickness). Details of AFM surface characterization of the samples are shown in Appendix B.

**Figure 1:**
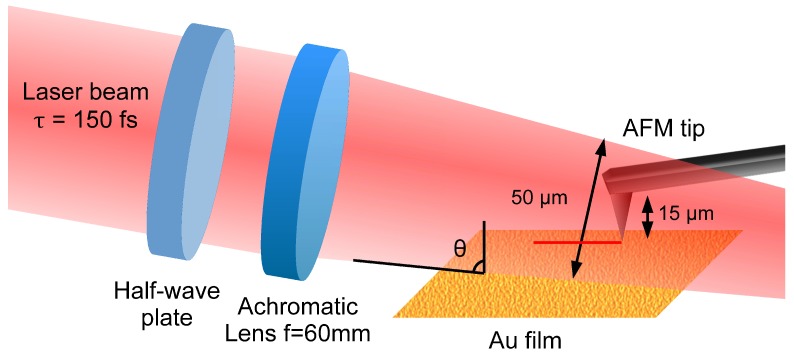
A scheme of the experimental setup. The p-polarized laser beam is focused onto an atomic force microscope (AFM) tip by an achromatic lens. The tip height is about 15 μm, so the laser focal spot size (50 μm) illuminates the tip entirely. A half-wave plate allows for controlling the laser polarization angle.

A scheme of the experimental setup is shown in Figure 1. The laser source for the experiments was a home-built femtosecond Ytterbium-doped fiber laser oscillator, designed according to [31]. The output of the laser cavity was directed to a pair of diffraction gratings that compensate the dispersion and compress the laser pulse. The laser beam consisted of laser pulses with temporal length τ = 150 fs, 1 nJ energy per pulse, central wavelength λ = 1040 nm, 46 MHz repetition rate, and linear polarization. The polarization was controlled by a half-wave plate. The laser power was adjusted by a combination of another half-wave plate and a polarizing beamsplitter cube. After passing through a periscope, the laser beam was focused onto the SPM tip by an achromatic lens (AC254-060-B-ML, Thorlabs, Newton, NJ, USA) with a focal length f = 60 mm. The laser spot diameter at the focal plane was 50 μm and the angle of incidence θ = 88${}^{\circ }$ with respect to the tip axis. Under this condition, the tip with a height of ca. 15 μm was entirely irradiated. The laser peak intensity was adjusted during the experiments from a minimum of *I* = 0.15 × 10${}^{8}$ W/cm${}^{2}$ to a maximum value of *I* = 2.90 × 10${}^{8}$ W/cm${}^{2}$.

The nanostructuring procedure consisted of three steps. First, an AFM scan of the sample surface was performed to obtain the topography before laser irradiation. The stage scanned one line along the fast axis (*y*-axis) and then moved along the slow axis (*x*-axis) to the next line position, where the scan along the fast axis was repeated. In the second step, we scanned the same area and, at a certain position of the slow scanning axis, the scanning in the slow direction (*x*-axis) was paused. Then, the laser beam was engaged and the tip was irradiated, while moving along the fast axis (*y*-axis). After a number of laser pulses, the laser beam was turned off and the scan continued. Finally, we scan again the same area to observe any change on the surface.

Near-field simulations were performed using a MNPBEM17 toolbox, which is based on a boundary element method (BEM) [33,34]. We introduced the values of the refractive indexes of silicon at 300 K [35] and 25 nm thick gold films [36]. The simulations were performed taking into account the retardation of the electromagnetic fields, by solving the full Maxwell equations.

## 3. Results

### 3.1. Near-Field Enhancement Simulations

**Figure 2:**
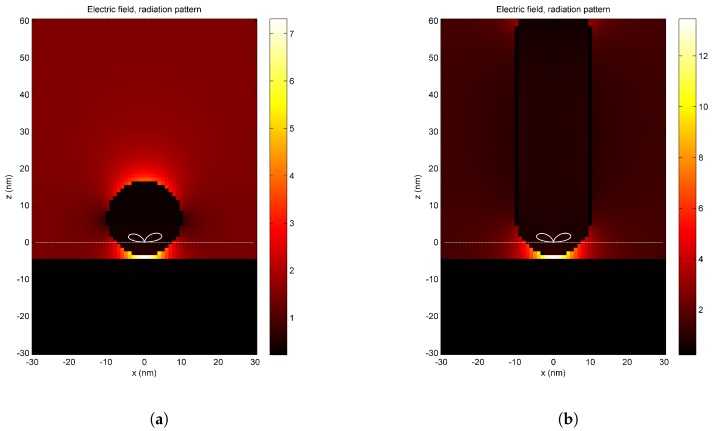
Near-field enhancement between a gold nanofilm and: (**a**) a spherical particle (radius = 10 nm); (**b**) a rod (r = 10 nm, length = 70 nm) both placed 1 nm above a gold surface. A p-polarized electromagnetic plane wave irradiates at λ = 1040 nm, with an angle of incidence θ = 88${}^{\circ }$. The white lines represent the direction and magnitude of the Poynting vector of far-field scattered laser light.

Near-field enhancement simulations were performed for a gold substrate and two silicon tip geometries, a sphere and a rod (Figure 2). The enhancement factor γ can be defined as γ = |*E*|/|*E*${}_{0}$|, where *E* is the induced electric field on the tip-substrate and *E*${}_{0}$ is the initial laser electric field. A p-polarized electromagnetic plane wave irradiated the tip at an angle of incidence θ = 88${}^{\circ }$ (referred to the axis normal to the substrate). The wavelength of the incoming laser light was set at λ = 1040 nm, which corresponds to the central wavelength of our fiber laser. We obtained near-field enhancement factors of γ ≈ 5–15 for the sphere, depending on the sphere’s radius of curvature and the tip-substrate distance (Figure 2a). The enhancement factor of the rod was higher than for the sphere and changed with the length L of the rod. Typical values ranged from γ = 12 (L = 70 nm, r = 10 nm) to γ = 220 (L = 200 nm, r = 10 nm) (Figure 2b). In both cases, the maximum field enhancement was located at the particle-substrate interspace and decreased exponentially with distance.

### 3.2. Scanning Probe Near-Field Nanolithography

In this section, we present the results of the laser irradiation and nanostructuring of gold nanofilms. In order to identify the irradiation parameters in the far-field which lead to irreversible modifications of the nanofilms, the threshold fluence as a function of the angle of incidence θ and the number of pulses *N* were determined (without the AFM tip engaged).

**Figure 3:**
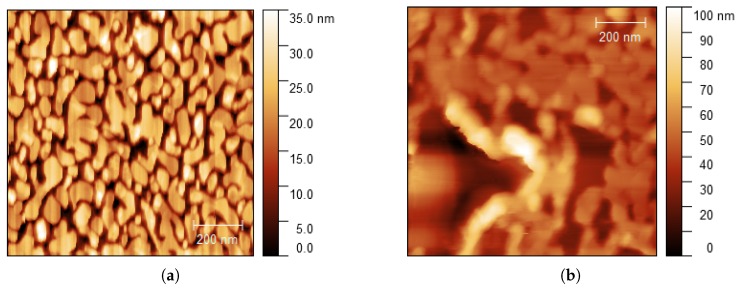
Morphology changes produced by the far-field laser. Gold on mica (15 nm thickness) (**a**) before laser irradiation; (**b**) after a laser irradiation time of 90 seconds, corresponding to *N* = 4.2 × 10${}^{9}$. Angle of incidence θ = 86${}^{\circ }$, *I* = 2.4 × 10${}^{8}$ W/cm${}^{2}$. Scan area 1 × 1 μm${}^{2}$.

The influence of the number of pulses *N* was studied on a 15 nm thick gold film on mica. An AFM image of the surface of the sample before laser irradiation can be seen on Figure 3a. The gold surface is composed of nanocrystalline islands with typical sizes of about 50–100 nm, similar to gold film surfaces reported in [37]. Far-field laser irradiation was performed at an angle of incidence of θ = 86${}^{\circ }$ and an intensity of *I* = 2.4 × 10${}^{8}$ W/cm${}^{2}$. The scanning direction was set from left to right, at a scanning speed of 1 μm/s. A substantial morphological modification was observed after an irradiation time of 90 seconds, corresponding to a number of pulses *N* = 4.2×10${}^{9}$ (Figure 3b). There, the gold film is strongly deformed and forms bumps that elevate to heights up to 100 nm. This experiment served to identify morphological surface changes produced by far-field irradiation.

**Figure 4:**
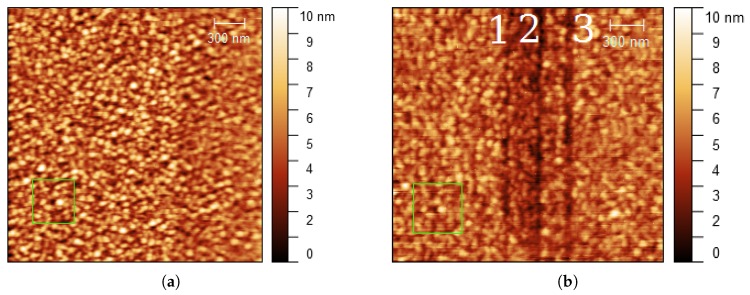
Nanolithography dependence on the number of scans at tip illumination. Gold on glass (30 nm thickness) (**a**) before laser irradiation and (**b**) after laser irradiation at three lines (slow axis scanning from left to right stopped). Number of scans: 7 (line 1), 10 (line 2) and 14 (line 3), scanning speed 0.62 μm/s, angle of incidence θ = 88${}^{\circ }$, *I* = 2.4 × 10${}^{8}$ W/cm${}^{2}$.

To analyze the influence of the angle of incidence, a similar experiment was conducted on a 30 nm thick gold film on glass at θ = 80${}^{\circ }$. Far-field surface modifications similar to Figure 3b were observed (not shown here), even for a lower number of pulses *N* = 0.19 × 10${}^{9}$. Therefore, we inferred that the angle of incidence has a drastic effect on far-field surface modifications, more significant than the number of pulses. In order to produce near-field nanolithography, far-field surface modifications need to be avoided. Therefore, the laser intensity was decreased by changing the angle of incidence to θ = 88${}^{\circ }$. At this angle of incidence, no far-field surface modifications were observed, even for a high number of pulses *N* = 4.2 × 10${}^{9}$. Figure 4a shows the surface of the sample (30 nm thick gold film on glass) before laser irradiation. Similar gold grain patterns can be observed before and after laser irradiation (e.g., green boxes in Figure 4a,b). This indicates that the far-field laser did not affect the surface morphology and only the areas below the tip were modified during irradiation.

Figure 4b shows the effect produced by repeated tip passes on the same line under laser illumination. The tip was irradiated at three lines (slow axis scanning stopped) during 30, 60 and 90 s (*N* = 1.2, 2.4 and 4.2 ×10${}^{9}$, respectively) (from left to right), corresponding to a number of tip passes on each line of 7, 10 and 14 times, respectively. Three vertical lines were structured on the gold surface (Figure 4b). The linewidth and depth both increased with the number of passes (Table 1).

**Figure 5:**
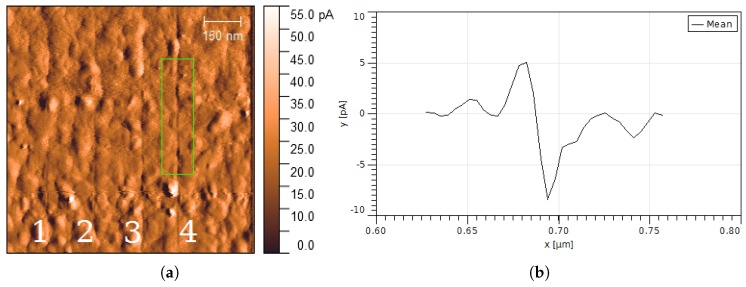
Nanolithography depending on laser intensity. Gold on mica (30 nm thickness) (**a**) after laser irradiation at four vertical lines (slow axis scanning from left to right stopped). *I* = 0.2, 0.3, 0.4, 1.0 × 10${}^{8}$ W/cm${}^{2}$, angle of incidence θ = 88${}^{\circ }$, scanning speed 0.38 μm/s; (**b**) averaged line profile of the area marked in Figure 5a. The FWHM width of the line is 10 nm. The image was obtained in AFM contact error mode, where a constant force is applied to the tip and the variations of the cantilever’s deflection are recorded.

The dependence on the laser intensity was investigated on a 30 nm thick gold film on mica (Figure 5a). The tip was irradiated at four vertical lines (slow axis scanning from left to right stopped) at increasing laser intensities *I* = 0.2, 0.3, 0.4, 1.0 × 10${}^{8}$ W/cm${}^{2}$ (from left to right). The scanning speed was set at 0.38 μm/s. Laser intensities below 0.4 ×10${}^{8}$ W/cm${}^{2}$ produced a very small effect. The depth of lines increased with the laser intensity. A line with an averaged FWHM width of 10 nm (Figure 5b) was produced at *I* = 1.0 × 10${}^{8}$ W/cm${}^{2}$. The line profile was obtained by averaging the green rectangle area marked in Figure 5a.

**Figure 6:**
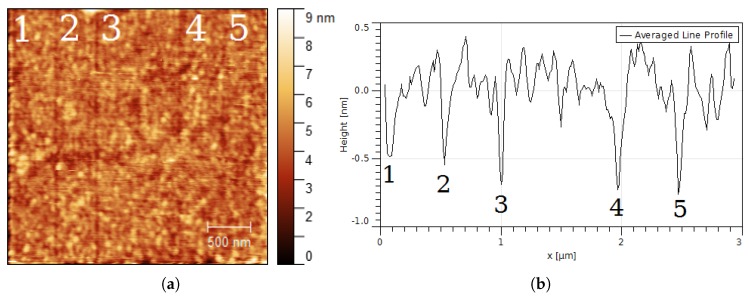
Nanolithography depending on the laser intensity. Gold on glass (30 nm thickness (**a**) after laser irradiation at five lines (slow axis scanning from left to right stopped). Laser intensity *I* = 0.7 (line 1), 1.0 (line 2), 2.0 (line 3), 2.7 (line 4) and 2.9 × 10${}^{8}$ W/cm${}^{2}$, scanning speed 0.38 μm/s, angle of incidence θ = 88${}^{\circ }$; (**b**) line profile obtained after taking an average of 256 horizontal profile lines. Widths and depths are indicated in Table 2.

Laser intensity dependence was also studied on a 30 nm thick gold film on glass. Figure 6a shows five vertical lines irradiated at increasing laser power *I* = 0.7, 1.0, 2.0, 2.7 and 2.9 × 10${}^{8}$ W/cm${}^{2}$ (from left to right) and scanning speed of 0.38 μm/s. The profile line was averaged (taking the full area in Figure 6a), to reduce the effect of the gold roughness. The FWHM width and depth of lines (Figure 6b) increased with the laser intensity (Table 2). The writing performance at lines 1, 4 and 5 was affected by 1–3 nm height irregularities of the gold surface.

A summary of the laser far-field parameters is provided in Table 3.

## 4. Discussion

Nanostructures were generated on the surface of gold films by using apertureless scanning near-field lithography. The structured lines formed on the gold films show typical linewidths of 40–70 nm and depths of 0.4–1.0 nm. The width of lines increased with the number of repeated tip scans and laser intensity. The smallest linewidth measured was 10 nm. The surface roughness of the gold samples affected the structuring performance (for instance, fourth and fifth lines in Figure 5). A threshold for surface modification at *I* ≈ 0.4 × 10${}^{8}$ W/cm${}^{2}$ (Figure 5a) was observed at a scanning speed of 0.38 μm/s. The angle of incidence (i.e., the laser intensity absorbed by the gold film) has a drastic effect on the far-field irradiation, as it can be seen in Figure 3b. At high angles of incidence (near 90${}^{\circ }$), the contrast between the near-field fluence near the tip and the far-field fluence on the illuminated substrate is sufficiently high so only nanostructures are generated without modification of the substrate (Figure 4b).

A comparatively low threshold fluence of *F*${}_{th}$ ≈ 6 μJ/cm${}^{2}$ for surface modification on gold was observed. This is in contrast to experiments with moderate repetition rates (1 kHz, *F*${}_{th}$ = 12 mJ/cm${}^{2}$) [4], with single pulses (*F*${}_{th}$ = 2 mJ/cm${}^{2}$) [38,39] and calculations with single pulses (*F*${}_{th}$ = 4.5 mJ/cm${}^{2}$) [40]. Near-field surface modification was also achieved at high repetition rates but lower energy per pulse (e.g., 80 MHz, 1 nJ) [8]. This seems to indicate that the repetition rate has a strong influence on the modification of the gold surface. The so-called *cool-ablation* [41] has been observed at very high repetition rates of GHz using bursts of laser pulses. Threshold fluences can be reduced when the repetition rate is increased above 1–10 MHz [41,42].

Two main mechanisms have been proposed in the context of apertureless scanning probe near-field lithography assisted by laser: near-field tip-enhancement and thermomechanical effects. Simulations in Figure 2 show the presence of an enhanced electromagnetic field between the tip and the substrate. The major question is whether this enhancement factor is high enough to raise the laser field above the modification threshold of the substrate. An experimental estimation of the near-field enhancement, based on the observation of far-field morphological surface changes of the gold films, was undertaken. In the experiments shown in the Figure 3, we observed evidence of complete melting of the gold film after a high number of pulses *N* = 4.2 × 10${}^{9}$ (Figure 3b). Based on this observation, the enhancement factor γ was determined by varying the angle of incidence on the 30 nm thick gold films. At an angle of incidence θ = 80${}^{\circ }$, the surface morphology was modified (not shown here), similarly to Figure 3b. Accordingly, the far-field fluence was above the modification threshold. An increase to an angle of incidence θ = 88${}^{\circ }$ resulted in a reduction of the absorbed laser energy by a factor of *I*${}_{88^{\circ }}$/*I*${}_{80^{\circ }}$ ≈ 0.02 (see Appendix A). Under these conditions, one observes near-field modification without far-field modification (see Figure 4). In a first approximation, one could conclude that the enhancement is at least *I*${}_{80^{\circ }}$/*I*${}_{88^{\circ }}$ ≈ 50.

This result is now compared with a simulated enhancement factor. For the spherical tip shown in Figure 2a, the electric field enhancement is γ ≈ 7. The intensity enhancement is $\gamma^{2}$ = |*E*${|}^{2}$/|*E*${}_{0}$${|}^{2}$ = 49, in good agreement with the intensity threshold obtained experimentally (*I*${}_{80^{\circ }}$/*I*${}_{88^{\circ }}$ ≈ 50).

## 5. Conclusions

Nanostructuring of gold films by apertureless scanning probe near-field lithography using a novel Ytterbium-doped fiber oscillator as the laser source was demonstrated. Lines written on gold show depths of 0.5–1.0 nm and typical lateral sizes of 40–70 nm, down to 10 nm. A near-field enhancement factor was determined experimentally and compared with simulations. A good agreement with the experimental results supports a mechanism based mainly on near-field enhancement. This approach provides direct access to the study of femtosecond laser-matter interaction in the near-field nano-regime. The compact setup, the high repetition rate, the possibility of working in ambient conditions and comparatively reduced cost make this an appealing technique for sub-100 nm lithography.

## Figures and Tables

**Table 1:** Dependence of width (FWHM – Full Width at Half Maximum) and depth of lines on the number of passes (in Figure 4b, from left to right). Data are averaged from 256 profile lines.

	Line 1	Line 2	Line 3
Number of passes	7	10	14
Width (nm)	36	45	76
Depth (nm)	0.4	0.5	1.0

**Table 2:** Laser intensity effect on width (FWHM) and depth of lines in Figure 6b (from left to right). Data are averaged from 256 profile lines.

Line Number	1	2	3	4	5
*I* (×10${}^{8}$W/cm${}^{2}$)	0.7	1.0	2.0	2.7	2.9
Width (nm)	71	47	41	60	52
Depth (nm)	0.48	0.52	0.70	0.74	0.77

**Table 3:** Far-field laser irradiation parameters for gold film on mica (15 nm thick) and on glass (30 nm thick): angle of incidence θ, laser peak intensity *I*, pulse fluence *F*, number of pulses *N*.

θ	*I* (×10${}^{8}$W/cm${}^{2}$)	*F* (μJ/cm${}^{2}$)	*N* (×10${}^{9}$)
80${}^{\circ }$	2.4	37	0.2
86${}^{\circ }$	2.4	37	1.4–4.2
88${}^{\circ }$	0.15–2.9	2–44	1.4–4.2

**Figure A1:**
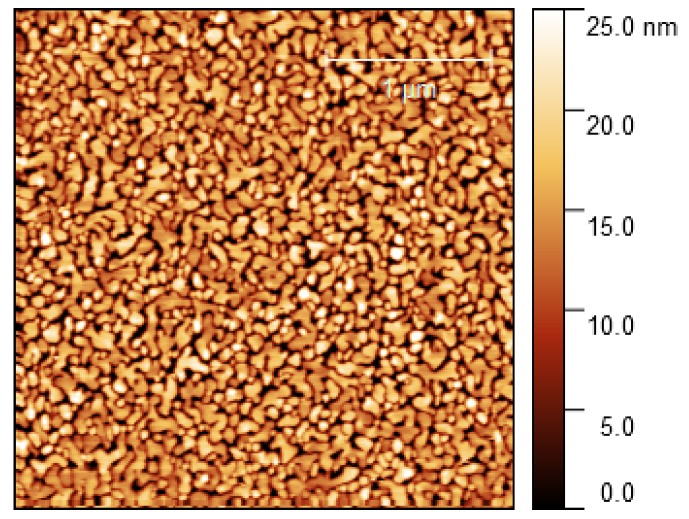
Topography of the 15 nm thickness Au film on mica imaged in AFM constant force mode; average surface roughness $R_{a}$ = 4.31 nm, scan size 3 × 3 μm${}^{2}$.

**Figure A2:**
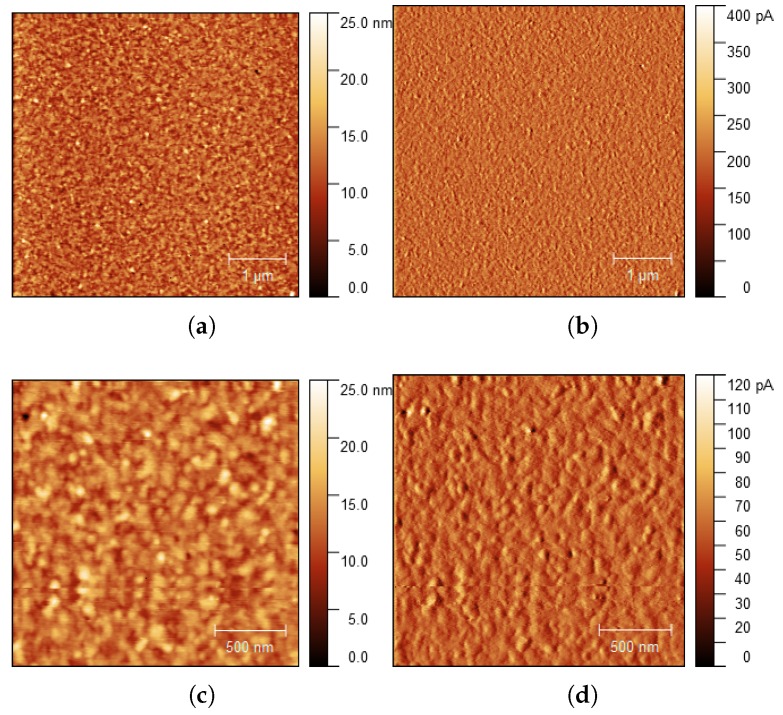
Topography of the 30 nm thickness Au film on mica imaged in AFM constant force mode (**a**,**c**) and error deflection mode (**b**,**d**). Average surface roughness $R_{a}$ = 1.71 nm (**a**), 20.84 pA (**b**), 1.75 nm (**c**) and 7.55 pA (**d**), scan size 5 × 5 μm${}^{2}$ (**a**,**b**) and 2 × 2 μm${}^{2}$ (**c**,**d**).

**Figure A3:**
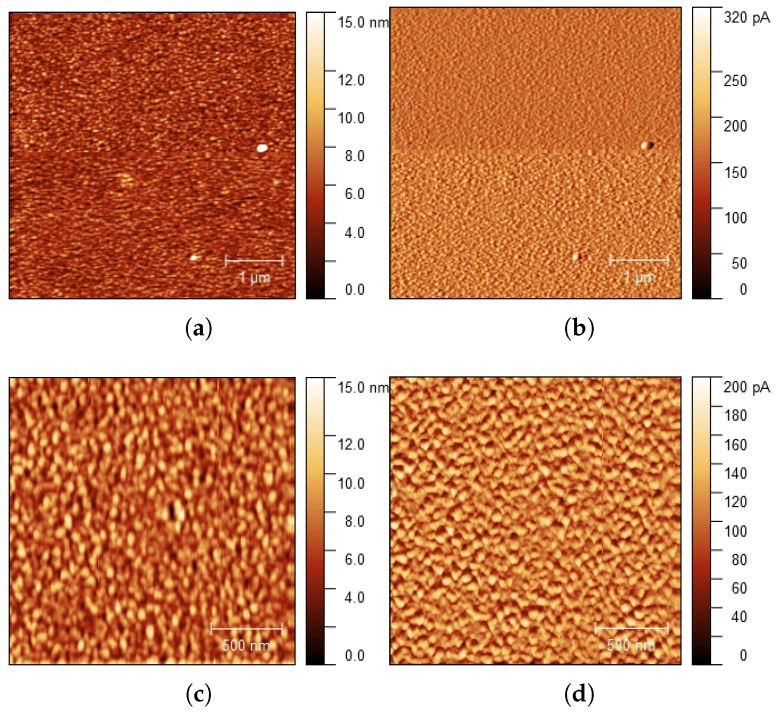
Topography of the 30 nm thickness Au film on glass imaged in AFM constant force mode (**a**,**c**) and error deflection mode (**b**,**d**). Average surface roughness $R_{a}$ = 1.12 nm (**a**), 18.88 pA (**b**), 1.53 nm (**c**) and 21.45 pA (**d**), scan size 5 × 5 μm${}^{2}$ (**a**,**b**) and 2 × 2 μm${}^{2}$ (**c**,**d**).

## Appendix A. Laser Absorbance Change versus Angle of Incidence for a Gold Nanofilm

The laser intensity threshold for surface modification was estimated by calculating the ratio between the absorbed laser intensity at two angles of incidence: θ = 80${}^{\circ }$ (far-field laser irradiation produced morphology changes of the gold surface) and 88${}^{\circ }$ (no far-field changes, only near-field when the tip was engaged). The two differences considered are the variation of both intensity and absorption with the incident angle. The first one can be calculated through the relationship

(A1) $$I\left(\theta \right)=I_{0}cos^{2}\left(\theta \right),$$

obtaining

(A2) $$\frac{I(80^{\circ })}{I(88^{\circ })}\approx 25.$$

Then, the change in the absorbed laser intensity by the gold surface at the two different angles of incidence can be calculated. By using the refractive index at λ = 1040 nm for 25 nm thick gold films [36] and the Fresnel equations for p-polarization, one can calculate the transmittance *T*

(A3) $$r_{p}=\frac{n_{2}cos\theta_{i}-n_{1}cos\theta_{t}}{n_{1}cos\theta_{t}+n_{2}cos\theta_{i}},$$

(A4) $$T=1-R=1-{\left|r_{p}\right|}^{2},$$

where $n_{1}$, $n_{2}$ are the refractive indexes of air and gold, $\theta_{i}$, $\theta_{t}$ are the incident and transmission angles, $r_{p}$ is the Fresnel reflection coefficient for p-polarization and *R* is the reflectivity. For simplicity, we assume that the transmitted laser light is completely absorbed by the gold substrate. This assumption is a good approximation because the penetration depth of light in gold ( δ≈ 12 nm at normal incidence ) is less than the gold film thickness. The transmittance values obtained at the angles of incidence considered are

(A5) $$\frac{T(80^{\circ })}{T(88^{\circ })}\approx 2.$$

A total absorbed intensity ratio is obtained by the multiplication of the ratios of Equations (A2) and (A5). This means that, when the angle of incidence is changed from θ = 80${}^{\circ }$ to θ = 88${}^{\circ }$, the absorbed laser intensity is reduced by a factor of 50.

## Appendix B. Morphology Characterization of the Surface of Au Films

The characterization of the topography of the Au films by AFM is presented here. Constant force and deflection error modes were recorded simultaneously by the AFM software (Nova, NT-MDT). Posterior analysis and calculation of average roughness values $R_{a}$ were performed with the software Gwyddion. The average roughness $R_{a}$ is defined as

(A6) $$R_{a}=\frac{1}{n}\sum_{i=1}^{n}\left|h_{i}\right|,$$

where *h* is the height of each pixel and *n* is the number of pixels. In addition, 256 × 256 pixels (full images) were included for the calculations of $R_{a}$.
